# Synopsis of the antimicrobial use guidelines for canine pyoderma by the International Society for Companion Animal Infectious Diseases (ISCAID)

**DOI:** 10.1111/vde.13365

**Published:** 2025-06-24

**Authors:** Anette Loeffler, Christine L. Cain, Lluís Ferrer, Koji Nishifuji, Katarina Varjonen, Mark G. Papich, Luca Guardabassi, Siân M. Frosini, Emi N. Barker, Scott J. Weese

**Affiliations:** ^1^ Department of Clinical Science and Services Royal Veterinary College Hatfield UK; ^2^ Department of Clinical Sciences and Advanced Medicine University of Pennsylvania, School of Veterinary Medicine Philadelphia Pennsylvania USA; ^3^ Department of Animal Medicine and Surgery Universitat Autònoma de Barcelona Barcelona Spain; ^4^ Division of Animal Life Science, Graduate School of Agriculture Tokyo University of Agriculture and Technology Tokyo Japan; ^5^ Anicura Albano Animal Hospital Danderyd Sweden; ^6^ College of Veterinary Medicine North Carolina State University Raleigh North Carolina USA; ^7^ Department of Veterinary and Animal Sciences University of Copenhagen Frederiksberg Denmark; ^8^ Langford Vets and Bristol Veterinary School University of Bristol Bristol UK; ^9^ Ontario Veterinary College University of Guelph Guelph Ontario Canada

## Abstract

**Background:**

Canine pyoderma is one of the most common presentations in small animal practice, frequently leading to antimicrobial prescribing.

**Objectives:**

To provide clinicians with antimicrobial treatment guidelines for staphylococcal pyoderma, including those involving meticillin‐resistant staphylococci. Guidance on diagnosing surface, superficial and deep pyoderma, and their underlying primary causes is included. Recommendations aim to optimise treatment outcomes while promoting responsible antimicrobial use.

**Materials and Methods:**

Evidence was gathered from a systematic literature review of English‐language treatment studies for canine pyoderma up to 23/12/2023. Quality was assessed using SORT criteria and combined with authors' consensus evaluation. Recommendations were voted on in an iterative process, followed by a Delphi‐style feedback process before final agreement by the authors.

**Results:**

Cytology should be performed in all cases before antimicrobials are used. Topical antimicrobial therapy alone is the treatment‐of‐choice for surface and superficial pyodermas. Systemic antimicrobials should be reserved for deep pyoderma and for superficial pyoderma when topical therapy is not effective. Systemic therapy, with adjunctive topical treatment, is initially provided for 2 weeks in superficial and 3 weeks in deep pyoderma, followed by re‐examination to assess progress and manage primary causes. First‐choice drugs have expected efficacy against the majority of meticillin‐susceptible *Staphylococcus pseudintermedius;* for all others, laboratory testing should confirm susceptibility and exclude suitability of safer alternatives. As culture and susceptibility testing are essential for rationalising systemic therapy, laboratories and practices should price them reasonably to encourage use. Proactive topical therapy using antiseptics may help prevent recurrences.

**Conclusions and Clinical Relevance:**

The accessibility of the skin offers excellent, achievable opportunities for antimicrobial stewardship.

## Abstract 2

**Background** – Canine pyoderma is one of the most common presentations in small animal practice, frequently leading to antimicrobial prescribing. **Objectives** – To provide clinicians with antimicrobial treatment guidelines for staphylococcal pyoderma, including those involving meticillin‐resistant staphylococci. Guidance on diagnosing surface, superficial and deep pyoderma, and their underlying primary causes is included. Recommendations aim to optimise treatment outcomes while promoting responsible antimicrobial use. **Conclusions and Clinical Relevance** – The accessibility of the skin offers excellent, achievable opportunities for antimicrobial stewardship.

## Zusammenfassung

**Hintergrund:**

Die canine Pyodermie ist eine der häufigsten Vorstellungsgründe in der Kleintierpraxis, was häufig zu einer Verschreibung von Antibiotika führt.

**Ziele:**

Das Ziel war es, KlinikerInnen mit Richtlinien zur antibiotischen Behandlung für eine Staphylokokken Pyodermie auszustatten, wobei auch Methicillin‐resistente Staphylokokken inkludiert waren. Die Anleitung eine Oberflächen‐, oberflächliche und tiefe Pyodermie sowie ihre zugrundeliegenden Primärursachen zu diagnostizieren ist inkludiert. Empfehlungen zielen darauf ab, Behandlungserfolge zu optimieren, indem ein verantwortungsbewusster Einsatz von Antibiotika befürwortet wird.

**Materialien und Methoden:**

Aus einer systematischen Literatur Review von Behandlungsstudien von caniner Pyodermie in englischer Sprache wurde bis 23/12/2023 Evidenz zusammengesucht. Die Qualität wurde mittels SORT‐Kriterien erfasst und mit der Consensus Evaluierung der AutorInnen kombiniert. Über die Empfehlungen wurde in einem schrittweisen Prozess abgestimmt, gefolgt von einem Delphi‐Stil Feedback Prozess vor der endgültigen Übereinstimmung der AutorInnen.

**Ergebnisse:**

In allen Fällen sollte vor Antibiotika Einsatz eine Zytologie durchgeführt werden. Eine alleinige topische antimikrobielle Therapie ist die Behandlung der Wahl für Oberflächen und oberflächliche Pyodermien. Eine systemische antimikrobielle Therapie sollte für tiefe Pyodermien reserviert werden sowie für oberflächliche Pyodermien, bei denen eine topische Therapie nicht wirksam war. Eine systemische Therapie mit zusätzlicher topischer Therapie sollte anfangs für 2 Wochen bei oberflächlicher und 3 Wochen bei tiefer Pyodermie eingesetzt werden, gefolgt von einer neuerlichen Untersuchung, um den Fortschritt zu erfassen und die Primärursachen zu managen. Medikamente der ersten Wahl haben eine erwartete Wirksamkeit gegenüber der Mehrheit der Methicillin‐sensiblen *Staphylococcus pseudintermedius*; für alle anderen sollten Laboruntersuchungen die Sensibilität bestätigen und den Einsatz sicherer Alternativen ausschließen. Nachdem bakterielle Untersuchungen und Antibiogramme essenziell sind, um die systemische Therapie vernünftig einzusetzen, sollten Laboratorien und Praxen diese zu vernünftigen Preisen anbieten, um ihren Einsatz zu unterstützen.

**Schlussfolgerungen und klinische Bedeutung:**

Die Zugangsmöglichkeit zur Hautoberfläche bietet exzellente, rationale und zielgerichtete Einsatzmöglichkeiten für Antibiotika.

## 摘要

**背景:**

犬脓皮症是小动物临床实践中最常见的疾病表现之一，常需要抗菌药物的使用。

**目的:**

为临床兽医提供葡萄球菌性脓皮症的抗菌治疗指南，包括涉及耐甲氧西林葡萄球菌的治疗建议。指南还包括表面性、浅表性和深层脓皮症的诊断方法及其潜在的原发病因。该指南旨在优化治疗效果，同时促进合理使用抗菌药物。

**材料与方法:**

通过系统性文献回顾，收集截至 2023年12月23日前英文发表的犬脓皮症治疗研究证据。采用 SORT 标准评估研究质量，并结合作者共识评估。推荐意见通过多轮投票，并采用 Delphi 风格反馈流程，最终由作者一致确认。

**结果:**

所有病例在使用抗菌药物前应进行细胞学检查。对于表面和浅表性脓皮症，单独的局部抗菌治疗为首选方案。全身性抗菌药物应仅用于深层脓皮症，或在浅表性脓皮症中外部治疗无效时使用。浅表性脓皮症的全身治疗应联合外部治疗，初始疗程为2周，深层脓皮症为3周，之后复查以评估疗效并处理原发病因。首选药物应对大多数对甲氧西林敏感的假中间型葡萄球菌有效；对于其他情况，实验室检测应确认药物敏感性，并排除更安全替代药物的可用性。由于培养和药敏测试对于合理制定系统治疗方案至关重要，实验室和诊所应合理定价，鼓励广泛使用。主动的外部抗菌治疗(使用抗菌剂)有助于预防复发。

**结论与临床意义:**

皮肤作为一个易接触的靶器官，为负责任的使用抗菌药物提供了良好且可实现的条件。

## Résumé

**Contexte:**

La pyodermite canine est l'une des affections les plus courantes chez les petits animaux, conduisant fréquemment à la prescription d'antimicrobiens.

**Objectifs:**

Fournir aux cliniciens des recommandations thérapeutiques pour le traitement antimicrobien de la pyodermite staphylococcique, y compris celle impliquant des staphylocoques résistants à la méthicilline. Des conseils sur le diagnostic de la pyodermite superficielle, profonde et de surface, ainsi que sur leurs causes primaires sous‐jacentes, sont inclus. Les recommandations visent à optimiser les résultats du traitement tout en encourageant une utilisation responsable des antimicrobiens.

**Matériels et méthodes:**

Les données ont été recueillies à partir d'une revue systématique de la littérature en anglais sur les études consacrées au traitement de la pyodermite canine jusqu'au 23/12/2023. La qualité a été évaluée à l'aide des critères SORT et combinée à l'évaluation consensuelle des auteurs. Les recommandations ont fait l'objet d'un vote dans le cadre d'un processus itératif, suivi d'un processus de retour d'information de type Delphi avant l'accord final des auteurs.

**Résultats:**

Une cytologie doit être réalisée dans tous les cas avant l'utilisation d'antimicrobiens. Le traitement antimicrobien topique seul est le traitement de choix pour les pyodermites superficielles et superficielles. Les antimicrobiens systémiques doivent être réservés aux pyodermites profondes et aux pyodermites superficielles lorsque le traitement topique n'est pas efficace. Le traitement systémique, associé à un traitement topique adjuvant, est initialement administré pendant 2 semaines pour les pyodermites superficielles et 3 semaines pour les pyodermites profondes, suivi d'un réexamen pour évaluer les progrès et traiter les causes primaires. Les médicaments de premier choix ont une efficacité attendue contre la majorité des *Staphylococcus pseudintermedius* sensibles à la méthicilline; pour tous les autres, des tests de laboratoire doivent confirmer la sensibilité et exclure la pertinence d'alternatives plus sûres. Comme les cultures et les tests de sensibilité sont essentiels pour rationaliser le traitement systémique, les laboratoires et les cabinets médicaux doivent les facturer à un prix raisonnable afin d'encourager leur utilisation. Un traitement topique proactif à base d'antiseptiques peut aider à prévenir les récidives.

**Conclusions et pertinence clinique:**

L'accessibilité de la peau offre d'excellentes possibilités réalisables en matière de gestion des antimicrobiens.

## 要約

**背景:**

犬の膿皮症は小動物診療において最も一般的な疾患の一つであり、しばしば抗菌薬の処方につながる。

**目的:**

メチシリン耐性ブドウ球菌を含むブドウ球菌性膿皮症に対する抗菌薬治療のガイドラインを臨床医に提供する。表面性膿皮症、表在性膿皮症、深在性膿皮症、およびそれらの根本的な主原因の診断に関するガイダンスを含む。推奨事項は、責任ある抗菌薬使用を促進しながら治療成績を最適化することを目的としている。

**材料と方法:**

2023年12月23日までの犬の膿皮症に関する英語による治療研究の系統的文献レビューからエビデンスを収集した。SORT基準を用いて質を評価し、著者のコンセンサス評価と組み合わせた。推奨は、著者による最終的な合意の前に、デルフィ式フィードバックプロセスを経て、反復プロセスで投票された。

**結果:**

抗菌薬を使用する前に、すべての症例で細胞診を実施すべきである。表面および表在性の膿皮症に対しては、局所抗菌薬療法のみを選択すべきである。抗菌薬の全身投与は、深在性膿皮症および局所療法が無効な場合の表在性膿皮症にのみ行う。表在性膿皮症では2週間、深在性膿皮症では3週間の全身療法および補助的な局所療法を行い、その後、経過を評価し、主原因を管理するために再検査を行う。第一選択薬は、メチシリン感受性*Staphylococcus pseudintermedius*の大部分に対して有効である。細菌培養検査および薬剤感受性検査は全身療法を合理化するために不可欠であるため、検査施設と診療所は適正な価格を設定して使用を奨励すべきである。消毒薬を用いた積極的な局所療法は、再発予防に役立つであろう。

**結論と臨床的意義:**

皮膚へのアクセスが容易であることは、抗菌薬スチュワードシップの優れた達成可能な機会を提供する。

## Resumo

**Contexto:**

A piodermite canina é uma das apresentações mais comuns na clínica de pequenos animais, frequentemente levando à prescrição de antimicrobianos.

**Objetivos:**

Fornecer aos clínicos diretrizes de tratamento antimicrobiano para piodermite estafilocócica, incluindo aquelas envolvendo estafilococos resistentes à meticilina. Orientações sobre o diagnóstico de piodermite superficial, profunda e superficial, e suas causas primárias subjacentes, estão incluídas. As recomendações visam otimizar os resultados do tratamento, promovendo o uso responsável de antimicrobianos.

**Materiais e Métodos:**

As evidências foram coletadas de uma revisão sistemática da literatura de estudos de tratamento em inglês para piodermite canina até 23/12/2023. A qualidade foi avaliada utilizando os critérios SORT e combinada com a avaliação consensual dos autores. As recomendações foram votadas em um processo iterativo, seguido por um processo de feedback no estilo Delphi antes da concordância final dos autores.

**Resultados:**

A citologia deve ser realizada em todos os casos antes do uso de antimicrobianos. A terapia antimicrobiana tópica isolada é o tratamento de escolha para piodermites de superfície e superficiais. Antimicrobianos sistêmicos devem ser reservados para piodermites profundas e para piodermites superficiais quando a terapia tópica não for eficaz. A terapia sistêmica, com tratamento tópico adjuvante, é inicialmente administrada por 2 semanas em piodermites superficiais e 3 semanas em piodermites profundas, seguida de reavaliação para acompanhar o progresso e controlar as causas primárias. Os medicamentos de primeira escolha têm eficácia esperada contra a maioria dos *Staphylococcus pseudintermedius* sensíveis à meticilina; para todos os outros, os testes laboratoriais devem confirmar a suscetibilidade e excluir a adequação de alternativas mais seguras. Como a cultura e os testes de suscetibilidade são essenciais para racionalizar a terapia sistêmica, laboratórios e clínicas devem precificá‐los de forma razoável para incentivar o uso. A terapia tópica proativa com antissépticos pode ajudar a prevenir recorrências.

**Conclusões e Relevância Clínica:**

A acessibilidade da pele oferece oportunidades excelentes e viáveis para a administração antimicrobiana.

## RESUMEN

**Introducción:**

La pioderma canina es una de las presentaciones más comunes en la práctica clínica de pequeños animales, lo que frecuentemente conlleva la prescripción de antimicrobianos.

**Objetivos:**

Proporcionar a los clínicos pautas de tratamiento antimicrobiano para la pioderma estafilocócica, incluyendo aquellas que involucran estafilococos resistentes a la meticilina. Se incluye orientación sobre el diagnóstico de pioderma externa, superficial y profunda, y sus causas primarias subyacentes. Las recomendaciones buscan optimizar los resultados del tratamiento, promoviendo al mismo tiempo el uso responsable de antimicrobianos.

**Materiales y métodos:**

La evidencia se obtuvo de una revisión sistemática de la literatura sobre estudios de tratamiento de pioderma canina en inglés hasta el 23/12/2023. La calidad se evaluó mediante los criterios SORT y se combinó con la evaluación por consenso de los autores. Las recomendaciones se votaron mediante un proceso iterativo, seguido de un proceso de retroalimentación tipo Delphi antes de la aprobación final por parte de los autores.

**Resultados:**

Se debe realizar una citología en todos los casos antes de utilizar antimicrobianos. La terapia antimicrobiana tópica por sí sola es el tratamiento de elección para las piodermas externas y superficiales. Los antimicrobianos sistémicos deben reservarse para las piodermas profundas y para las piodermas superficiales cuando la terapia tópica no es eficaz. La terapia sistémica, con tratamiento tópico adyuvante, se administra inicialmente durante 2 semanas en las piodermas superficiales y 3 semanas en las profundas, seguida de una reevaluación para determinar la evolución y abordar las causas primarias. Los fármacos de primera elección tienen la eficacia esperada contra la mayoría de los *Staphylococcus pseudintermedius* sensibles a la meticilina; para todos los demás, las pruebas de laboratorio deben confirmar la sensibilidad y descartar la idoneidad de alternativas más seguras. Dado que el cultivo y las pruebas de sensibilidad son esenciales para racionalizar la terapia sistémica, los laboratorios y las clínicas deben fijar un precio razonable para fomentar su uso. La terapia tópica proactiva con antisépticos puede ayudar a prevenir las recurrencias.

**Conclusiones y relevancia clínica:**

La accesibilidad de la piel ofrece excelentes oportunidades para la optimización del uso de antimicrobianos.

## BACKGROUND

Pyoderma (bacterial skin infection) is common in dogs and often leads to antimicrobial prescribing. This synopsis presents the consensus statements from the new antimicrobial use guidelines for canine pyoderma, together with some brief context on the management of canine pyoderma. The full document, including the underpinning literature reviews and evidence is available as an open‐access document at https://doi.org/10.1111/vde.13342.^1^

## TYPES OF CANINE PYODERMA AND BACTERIAL PATHOGENS

**FIGURE 1:**
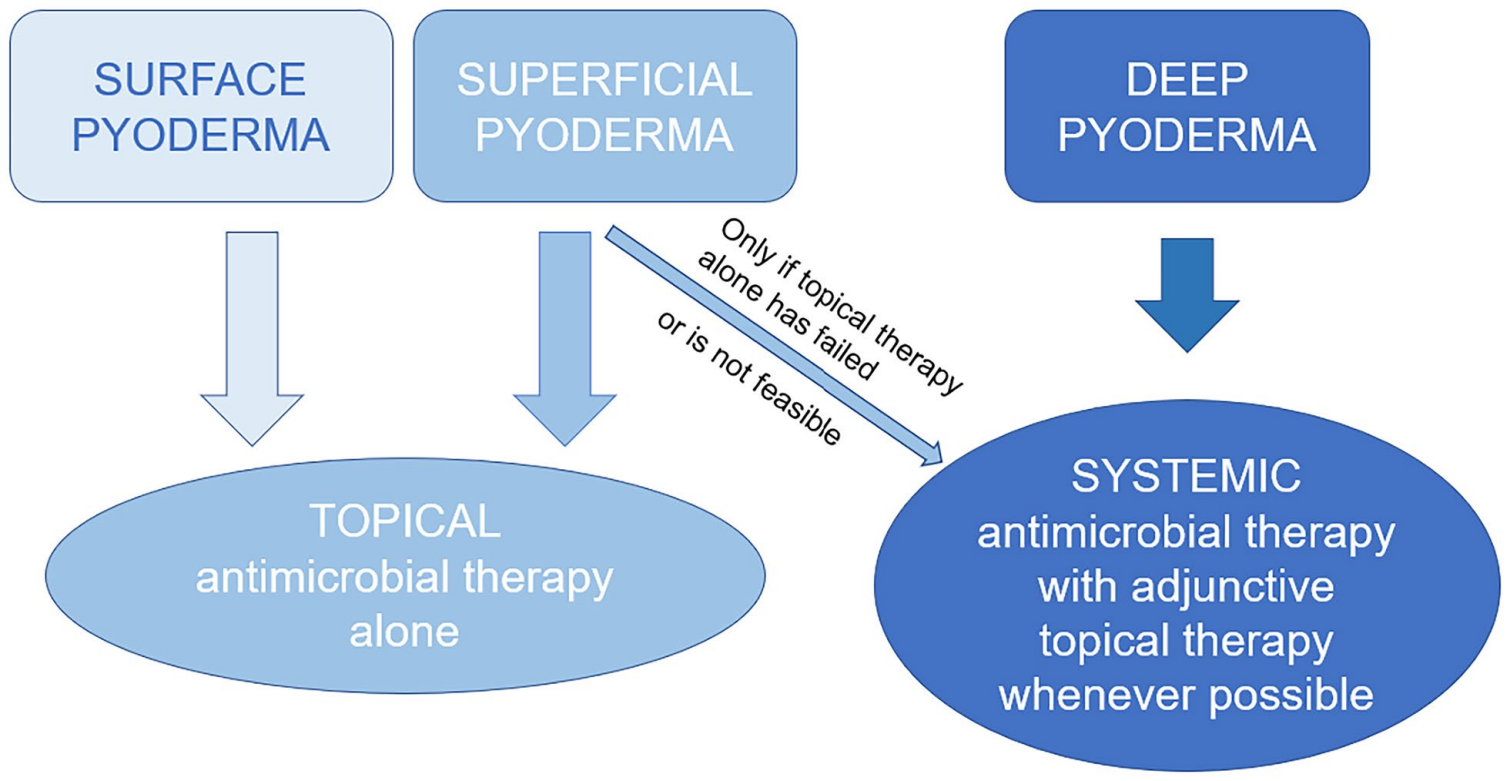
Histopathological classification of canine pyoderma by depth, and recommended treatment approaches (topical antimicrobial therapy including antiseptics and topical antibiotics).

For the purpose of making treatment recommendations, the classification of pyoderma by histological depth of infection has been adopted (Figure 1).

Surface, superficial and deep pyoderma are differentiated clinically by recognising lesion types and locations on the body (Table 1).

**TABLE 1:** Commonly recognised presentations of canine pyoderma by depth of infection as used in these guidelines and examples of typical lesions and findings.

Depth/type of pyoderma	Clinical presentations	Most common lesions and frequent clinical findings
Surface	Pyotraumatic (acute moist dermatitis (“hot spot”) Intertrigo (skin‐fold dermatitis) Bacterial overgrowth syndrome	Erythema, exudation, alopecia, pruritus Erythema, malodour Erythema, surface exudate
Superficial	Superficial bacterial folliculitis (SBF) Exfoliative superficial (spreading) pyoderma Impetigo Mucocutaneous pyoderma	Papules, follicular pustules, epidermal collarettes, crusts Epidermal collarettes, scales, crust Papules, interfollicular pustules, epidermal collarettes Erythema, crusting, hypopigmentation
Deep	Widespread furunculosis/cellulitis Localised furunculosis/cellulitis, for example: ◦ Pyotraumatic folliculitis and furunculosis ◦ Acral lick dermatitis ◦ Infected interdigital nodules ◦ Callus pyoderma ◦ Chin pyoderma/chin ‘acne’ Postgrooming furunculosis	Can be present in any presentation of deep pyoderma: Haemorrhagic crusts, haemopurulent discharge, fistulae (sinuses), ulcers, nodules, plaque, ill‐defined swelling Pain, lymphadenomegaly

*Staphylococcus pseudintermedius* is isolated from over 90% of superficial pyoderma and around 60% of deep pyoderma cases. A more mixed microbial population can be expected from surface pyoderma.

## DIAGNOSTIC APPROACH AND TESTS

**FIGURE 2:**
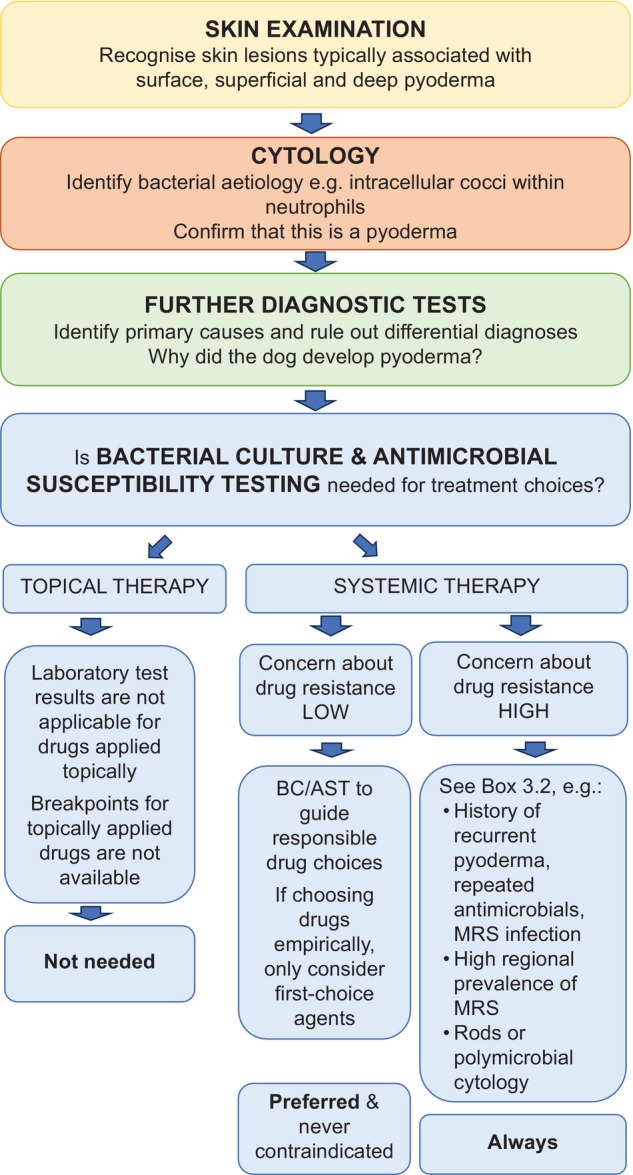
Stepwise diagnostic approach to a dog with pyoderma.

A 3‐step approach to every case of suspected pyoderma is recommended before prescribing antimicrobial treatment (Figure 2).

The skin should be examined in its entirety to determine lesion type and distribution in order to identify the type of pyoderma.

Cytology from affected skin should be performed in every case of suspected canine pyoderma to confirm a bacterial cause.

Cytology can be performed in‐house with immediate results and is critical to differentiate bacterial causes from sterile or fungal aetiologies.^2^

Our group agreed that the following findings are supportive of pyoderma:

- Intracellular bacteria *or*
- Extracellular bacteria with nuclear streaking (also called streaming) *or*
- As in the case of bacterial overgrowth, large numbers of bacteria from lesional skin in the absence of inflammatory cells.

Pyoderma is always secondary to underlying primary causes, and these must be considered at the first occurrence.

Trauma (e.g., abrasions, cuts, friction, chronic pressure), ectoparasitic infestation and atopic skin disease are the most common primary underlying diseases for pyoderma, but altered cornification and/or systemic diseases such as endocrinopathies and neoplasia may also need to be considered. Appropriate diagnostic tests need to be chosen depending on historical and clinical findings.

Bacterial culture and antimicrobial susceptibility testing (BC/AST) is recommended to guide drug choices whenever systemic therapy is planned and is preferred over empirical choices.

BC/AST is always strongly recommended when there is an increased risk of resistance to common empirically chosen antimicrobials, for example if there is a history of recent or frequent antimicrobial use, of previous isolation of meticillin‐resistant *Staphylococcus pseudintermedius* (MRSP), *S. aureus* (MRSA) or *S. coagulans* (MRSC) (formerly *S. schleiferi*), or in regions or clinics with a high prevalence of meticillin‐resistance.

BC/AST should always be paired with cytology to guide correct interpretation of laboratory results.

Samples should be submitted to laboratories that follow recognised standards for BC/AST and use breakpoints for pathogens isolated from veterinary species, currently only published by the Clinical and Laboratory Standards Institute (CLSI).^3^ Where multiple bacterial isolates are reported, treatment should be targeted against the predominant pathogen as supported by the cytology findings, in most cases *S. pseudintermedius*. The best sampling technique will vary depending on lesion type and sampling purpose (Table 2).

**TABLE 2:** Tips for sampling of skin lesions for cytological evaluation and bacterial culture.

Lesion type	Comment	For cytological evaluation	For bacterial culture
Pustule (or bulla)	Pustule content optimal owing to low risk of contamination with commensals	Lance pustule with sterile needle, transfer content directly onto glass slide (impression smear)	No surface disinfection. Lance with sterile needle and transfer pus (or haemopurulent material) to sterile swab
Pus (exudative lesions)	From skin surface or expressed from draining tracts/sinuses. Remove dried surface material before sampling	Impression smear directly onto slide or use swab to sample, then roll onto slide	Squeeze and discard surface pus using a single wipe with 70% alcohol. Allow alcohol to dry, then squeeze again and sample using a sterile swab
Crust, epidermal collarette with peripheral crusting	Underside of crust or skin below is expected to yield representative bacteria	Lift crust using sterile needle, sample exposed skin with swab, slide or tape	Lift crust using sterile forceps or needle. Sample the exposed exudate or skin or beneath the “leading edge” of an epidermal collarette with a sterile swab
Papule	Papule content is preferrable to surrounding skin	Pinch skin to express fluid or blood or tape strip to lift material surrounding the papule	Pinch the skin to express fluid or blood onto a sterile swab; surface sampling may be unrewarding
Dry lesions (old epidermal collarettes)	Border of epidermal collarettes preferred over centre of lesion	Tape strip to lift diagnostic material or roll/rub swab two to three times over lesion, then smear on slide	No surface disinfection. Roll sterile swab beneath the leading edge / across the border of the collarette two to three times
Erythematous skin	When no other lesions are found or when surface pyoderma is suspected (e.g. intertrigo)	Tape strip sampling or rotate swab several times over affected skin (or within a fold), then roll onto slide	Rarely needed
Deep lesions	For example interdigital nodules or plaques	Needle aspirates or tissue impression after biopsy	Provide sedation, local or general anaesthesia as appropriate. Clip hair, clean and disinfect surface (wipe with 70% alcohol, let dry before sampling). Consider wearing gloves. Obtain tissue by biopsy (or fine needle aspirate) and submit in a sterile container or bacterial transport medium

## SURFACE PYODERMA

**FIGURE 3:**
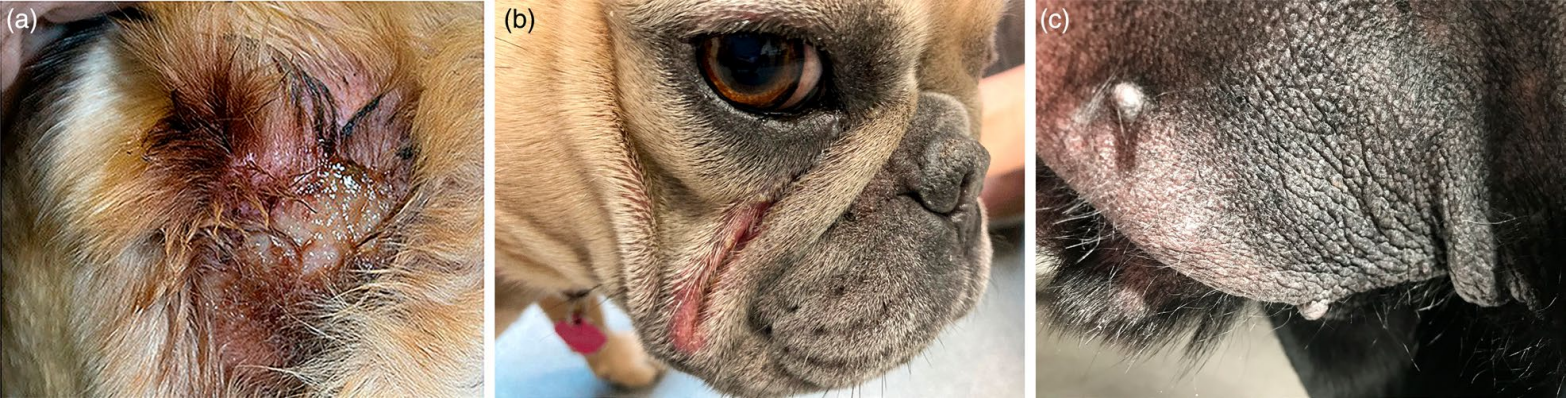
Examples of surface pyoderma. (a) Pyotraumatic dermatitis (‘hot spot’) on the lateral neck of a Golden retriever (image courtesy S. Shaw). (b) Intertrigo (skin fold dermatitis) affecting the facial folds of a bulldog. (c) Bacterial overgrowth syndrome on the ventral abdomen of a Labrador retriever.

Surface pyoderma encompasses **pyotraumatic dermatitis** (‘acute moist dermatitis, hot spot’), **intertrigo** (fold dermatitis) and **bacterial overgrowth syndrome** (BOG) (Figure 3).

### Treatment recommendations for surface pyoderma

Topical antimicrobial therapy is the treatment of choice for surface pyoderma.

Several studies have shown good clinical efficacy (reductions in lesion and bacterial scores) of topical antimicrobial therapy alone. Clinical resolution of surface pyoderma can be assumed when pre‐treatment lesions have significantly improved or resolved and cytology from previously affected areas is normal. This can be expected within 7–14 days. If progress is deemed poor by the owner, re‐assessment of clinical signs by a veterinarian and repeat cytology are recommended.

Combination therapy of topical antimicrobial therapy with topical glucocorticoids or with a short course (5–7 days) of systemic glucocorticoids at anti‐inflammatory doses or with antipruritic medication may be helpful in cases of pyotraumatic dermatitis and of intertrigo where an inflammatory or pruritic primary cause is involved.

Inflammation likely plays a major role in surface pyoderma. Good efficacy of adjunctive anti‐inflammatory medication in the management of surface pyoderma was shown in several clinical trials.

Antiseptic treatment can be continued proactively on previously affected skin, potentially life‐long, where the primary underlying causes cannot be resolved (e.g. skin folds) and the risk of recurrence remains.

Frequency of application is determined on a case‐by‐case basis, but daily or alternate day applications are typically required initially to achieve remission. Depending on the clinical presentation, effective preventative measures can also include weight loss or surgical interventions for skin folds, or in many cases proactive therapy of underlying allergic disease with topical anti‐inflammatory therapy. Analgesia should be considered, particularly in pyotraumatic dermatitis, which is often painful.

## SUPERFICIAL PYODERMA

**FIGURE 4:**
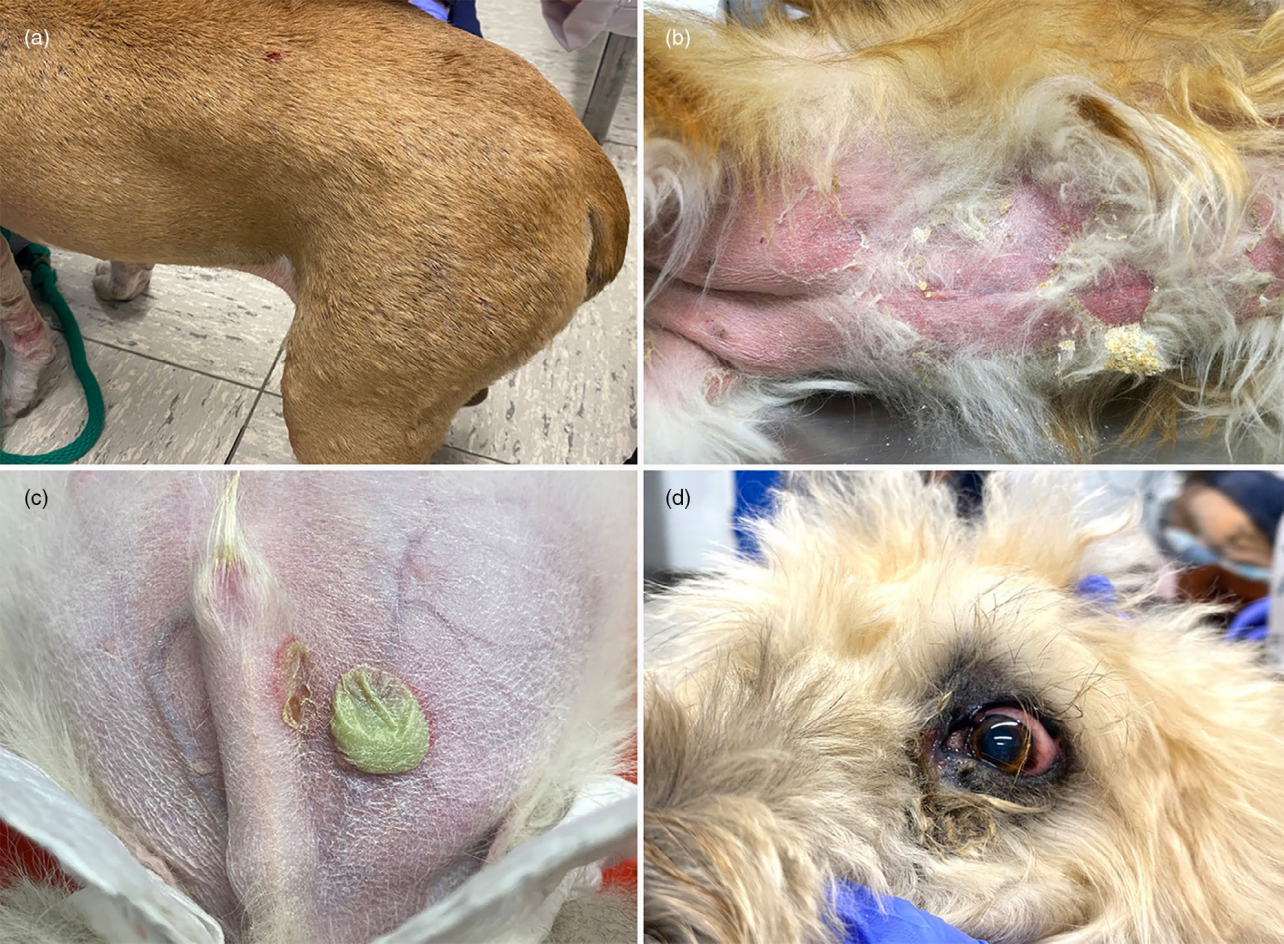
Examples of superficial pyoderma. (a) Superficial bacterial folliculitis on the trunk and lateral thigh of an American pitbull terrier. (b) Exfoliative superficial pyoderma in a Shetland sheepdog. (c) Impetigo on the ventral abdomen of a Siberian husky puppy. (d) Mucocutaneous pyoderma on the eyelid margin and medial canthus in a soft‐coated Wheaten terrier.

Superficial pyoderma includes **superficial bacterial folliculitis (SBF)** as the most common type of superficial pyoderma in the dog, **exfoliative superficial pyoderma** (previously termed ‘superficial spreading pyoderma’), **impetigo** and **bullous impetigo** and **mucocutaneous pyoderma** (Figure 4).

### Treatment recommendations for superficial pyoderma

Topical antimicrobial therapy as the sole antibacterial treatment is the treatment of choice for canine superficial bacterial folliculitis and for other presentations of superficial pyoderma.

Response to topical therapy should be reassessed by a veterinarian after 2–3 weeks.

Topical antimicrobial therapy as the sole antibacterial treatment has been found effective in 12 of the 13 published clinical trials, including eight randomised trials and one study confirming that the efficacy of topical therapy alone was no different to that of systemic therapy. Lesion improvement was noticeable in most dogs within 1–2 weeks, resolution within 3–4 weeks. Topical treatment is then maintained until all lesions are resolved *and* underlying primary causes identified and addressed.

Systemic antimicrobial therapy should be reserved for cases that have failed to respond to topical antimicrobial therapy alone or if topical therapy is not feasible due to client or patient limitations.

There are no clinical reasons or indications that would justify the selection of systemic over topical therapy, but if the dog's temperament, owner's ability or compliance, or available facilities make topical treatment impossible, or if lesions have not improved sufficiently after 2 weeks of topical therapy alone, systemic antimicrobials will need to be considered.

BC/AST and cytology should be used whenever possible to guide systemic drug choices.

Drugs should only be chosen empirically when the risk for meticillin‐resistance is deemed low and only first‐choice agents (clindamycin, cefalexin, cefadroxil, amoxicillin‐clavulanate) should be considered for empirical selection.

An initial 2‐week course of systemic antimicrobials may be dispensed and an appointment for re‐examination by a veterinarian should be scheduled prior to the end of the course to determine whether systemic treatment can be stopped or whether longer treatment is required.

Where a re‐examination before the end of the initial 2‐week course is not feasible, treatment can be extended to avoid stopping treatment before veterinary assessment.

Adjunctive topical antimicrobial therapy is recommended whenever possible.

Clinical resolution of superficial pyoderma can be assumed, and systemic antimicrobials stopped when primary lesions of pyoderma (papules, pustules and erythematous epidermal collarettes) are no longer found.

There is no evidence to support extending systemic antimicrobial therapy beyond the resolution of clinical signs associated with infection; instead, underlying primary causes must be identified and addressed.

While not all cases of superficial pyoderma resolve within 2 weeks, many did based on our literature review. Combining a shorter initial course with adjunctive topical antimicrobial therapy and scheduled re‐examinations provides opportunities for further diagnostics of underlying primary conditions, and for early detection of drug resistance if progress is poor.

Topical antiseptic treatment can be continued longer than systemic therapy, potentially life‐long, where the primary causes cannot be resolved and the risk of recurrence remains.

Little published information is available on the treatment of impetigo, exfoliative superficial pyoderma and mucocutaneous pyoderma, but topical antimicrobial therapy alone is recommended as an initial treatment approach, followed by assessment of response, treatment adjustments and investigation of primary disease as appropriate for the case.

## DEEP PYODERMA

**FIGURE 5:**
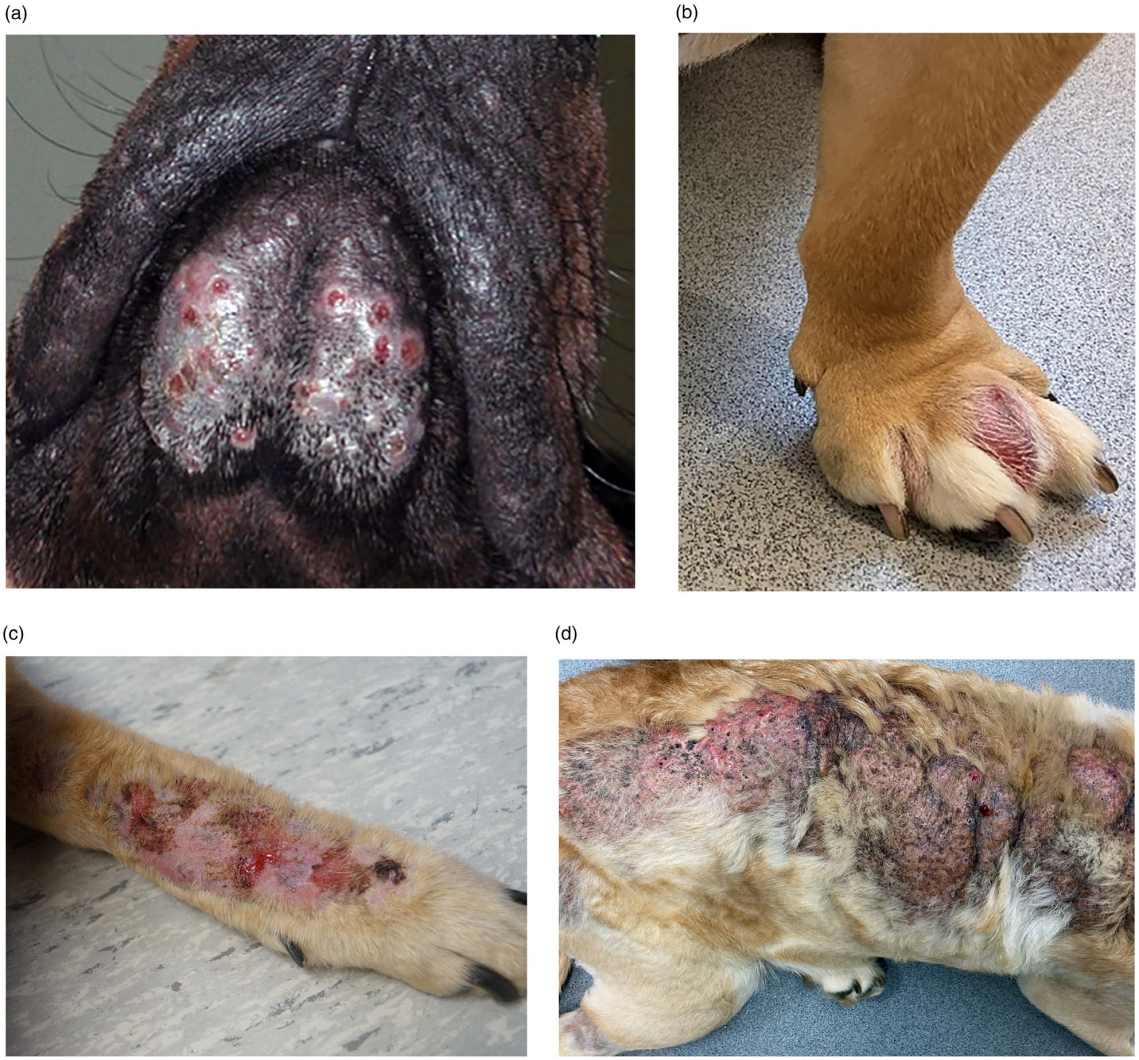
Examples of deep pyoderma in dogs. (a) Chin pyoderma (‘chin acne’). (b) Interdigital infected nodule. (c) Acral lick dermatitis. (d) Widespread deep folliculitis and furunculosis.

Deep pyoderma can be localised or widespread and includes a heterogeneous group of different clinical presentations such as deep folliculitis and furunculosis, postgrooming furunculosis, acral lick dermatitis, interdigital infected nodules, pressure point/callus and chin pyoderma (Figure 5). Deep pyoderma is potentially serious, painful or debilitating with a risk of haematogenous spread and progression to septicaemia.

### Treatment recommendations for deep pyoderma

Systemic antibacterial therapy is always indicated in deep pyoderma and the choice of drug should always be based on BC/AST results.

Systemic antimicrobials are best started when BC/AST results are available. However, if there is a high risk of deterioration or septicaemia, empirical treatment guided by cytology findings can be started immediately with re‐evaluation of drug selection when laboratory results are received.

Adjunctive topical antimicrobial therapy is recommended in every case as soon as the dog is considered pain‐free.

Although good evidence is lacking, topical therapy is expected to facilitate healing by removing crust, reducing microbial load on the surface, and possibly shortening the duration of systemic therapy required.

An initial 3‐week course may be dispensed, and an appointment for re‐examination by a veterinarian should be scheduled prior to the end of the course to determine whether systemic treatment can be stopped or whether longer treatment is required.

If clinical signs are improving but have not resolved and if cytological evidence of infection is still present, treatment should be continued with re‐evaluation every 2 weeks.

Systemic antimicrobial therapy can be stopped when skin lesions associated with deep infection (draining / fistulous tracts, pus, pustules, crusts) have resolved and there is no cytological evidence of infection.

At re‐examination, if draining/fistulous tracts, pus, pustules, ulcers, or crusts remain or if lesions are no longer improving (have plateaued), their bacterial nature should be confirmed again by cytology to differentiate ongoing infection from sterile disease processes; if bacteria are seen on cytology, BC/AST need to be repeated to investigate drug resistance.

Topical antiseptic treatment can be continued beyond stopping systemic therapy where the primary underlying cause(s) cannot be resolved and the risk of recurrence remains.

There is no evidence to support extending systemic antimicrobial therapy beyond the resolution of clinical signs; instead, underlying primary causes must be identified and addressed.

A shorter initial course of 3 weeks is recommended, supported by adjunctive topical antimicrobials and scheduled re‐examinations every 2 weeks to monitor progress. It should be very rare that more than 6 weeks of appropriate antimicrobial therapy is required to eliminate the secondary pyoderma component of skin lesions.

Medication to relieve pain should be considered if the dog is likely to be in pain. Measures such as adjunctive fluorescence biomodulation, whirlpool therapy, and weight loss can be supportive in individual cases but evidence for efficacy is sparse.

## TOPICAL ANTIMICROBIAL THERAPY

Topical therapy used as sole antimicrobial treatment is the treatment of choice for all cases of surface and superficial pyoderma and should also be considered as adjunct therapy in all cases of pyoderma that require systemic treatment.

Antiseptics (e.g. chlorhexidine) should be prioritised over topically used antibiotics (e.g. fusidic acid, mupirocin). To improve the efficacy of topical antimicrobial therapy, the removal of surface debris and the physical disruption of potential biofilms using water and wipes or shampoos may be helpful.

Currently, the agent with the greatest support for efficacy in canine pyoderma is chlorhexidine at 2–4%, available and found effective in several compositions and formulations (surgical scrub, shampoo, solution). However, in vitro evidence suggests that low‐concentration chlorhexidine products may have reduced efficacy against some Gram‐negative bacteria. Other agents such as silver sulfadiazine might need to be considered if rods predominate on cytology. Good‐quality evidence for efficacy also was found for a chlorhexidine/miconazole combination, benzoyl peroxide, olanexidine and a dilute sodium hypochlorite (NaOCl; bleach) / salicylic acid combination product. Although chlorine is an excellent bactericidal agent, in the form of household bleach, typically sold as 3%–8% at the time of manufacturing, it requires significant dilution to achieve a concentration considered safe for use on dogs and for owners (<0.05%).

Fusidic acid and mupirocin are known for their narrow‐spectrum anti‐staphylococcal activity and are available for topical therapy. Although there are currently no clinical data on fusidic acid when used alone in canine pyoderma, its efficacy when used as a topical combination product with betamethasone was shown to be no different to that of systemic amoxicillin/clavulanic acid and dexamethasone in dogs with pyotraumatic dermatitis. In one abstract presentation, clinical efficacy of a 0.2% mupirocin spray was suggested to be comparable to a 4% chlorhexidine mousse, when both were applied twice daily for 3 weeks, in reducing lesion and pruritus scores in five dogs with pyoderma (depth not stated). Many other molecules (antiseptics and topical antibiotics) have had efficacy shown against staphylococci in vitro; however, owing to a lack of strong clinical evidence for efficacy, these agents are not included in recommendations at present. For some localised, inflamed lesions, for example in surface pyodermas, topical antibiotics may be advocated and effective in the form of multi‐pharma ear drop formulations (off‐licence).

Topical antimicrobial formulations can be considered safe if used according to manufacturers' or literature‐recommended instructions and provided no known hypersensitivities are reported. True antimicrobial resistance resulting in clinical treatment failure of staphylococcal infection using chlorhexidine has not yet been described, and MICs of staphylococci from dogs have been shown to be low against various topical antimicrobials.

## SYSTEMIC ANTIMICROBIAL THERAPY FOR CANINE PYODERMA

Drugs appropriate for canine pyoderma were assigned to four groups following considerations of efficacy and safety based on published evidence and importance of a drug in human medicine. Recommended dosages may deviate from previous recommendations for staphylococcal skin infections and from registration datasheets. Updates are based on a careful review of the current approved label recommendations in various countries, updated PK‐PD analysis, and the dose that was used to determine approved susceptibility testing. It is the responsibility of the prescribing veterinarian to be familiar with national prescribing regulations relating to antimicrobials for use in dogs. General concepts such as correct weighing of dogs and reminding owners of the need for good compliance with dosage instructions are important.

### Empirical versus culture‐based drug‐choices

Empirical systemic antimicrobial therapy should only be considered where the risk for drug resistance is low.

Only first‐choice drugs (clindamycin, cefalexin, cefadroxil, amoxicillin‐clavulanate) should be considered for empirical therapy.

First‐choice drugs are expected to have good efficacy against MSSP.

Because cost (and occasionally practicality) is likely to be the main driver for empirical selection of drugs, owners should be made aware that the cost of antimicrobial resistance may ultimately exceed that of BC/AST should empirically chosen drugs turn out to be ineffective; likewise, practices and laboratories should support antimicrobial stewardship by charging reasonably for BC/AST.

### First‐choice drugs

Representatives are expected to be available and approved in most countries, and susceptibility testing breakpoints for staphylococci from dogs are available for all except lincomycin (Table 3).

**TABLE 3:** ‘First‐choice’ antimicrobial drugs for canine pyoderma.

First‐choice drugs: Good, predicted efficacy against most meticillin‐susceptible *Staphylococcus* spp., low risk of adverse effects		
**Drug**	**Suggested dosage**	**Comments**
Amoxicillin‐clavulanate acid	12.5 mg/kg p.o. q12h	Oral penicillin‐beta‐lactamase inhibitor Can increase faecal shedding of resistant *E.coli* during treatment
Cefalexin, cefadroxil	22–25 mg/kg p.o. q12h	Oral first‐generation cephalosporins Can increase faecal shedding of resistant *E.coli* during treatment
Clindamycin	11 mg/kg p.o. q12h	Although there is evidence for successful treatment with 5.5 mg/kg q12h, we recommend a higher dose for a more consistent clinical response
Lincomycin	22 mg/kg p.o. q12h	Insufficient evidence for or against use of lincomycin Oral forms for dogs may not be available in some regions

*Note*: Colours are critically important to highlight the different drug groups according to the antimicrobial group traffic light system.

Abbreviations: p.o., per os (administered orally); q12h, twice daily every 12 h.

### Second‐choice drugs

Drugs listed as ‘second‐choice’ should *only* be considered (i) when the causative bacterium is susceptible based on BC/AST results, *and* (ii) when first‐choice agents are not appropriate (Table 4). They either have an increased relative risk for the selection of important multidrug‐resistant pathogens or an increased risk of adverse health events.

**TABLE 4:** ‘Second‐choice’ antimicrobial drugs for canine pyoderma.

Second‐choice drugs: Only to be considered when bacterial culture and antimicrobial susceptibility test results are available, and when first‐choice drugs are not suitable		
Cefovecin	8 mg/kg subcutaneously once	Injectable third‐generation cephalosporin Can be repeated after 14 days for staphylococci Can be considered when compliance failure is a factor in achieving cure Restricted by regulatory authorities in some countries
Cefpodoxime proxetil	5–10 mg/kg p.o. q24h	Oral third‐generation cephalosporin Consistently active against meticillin‐susceptible *Staphylococcus* spp. Availability and usage may be geographically restricted
Enrofloxacin	5–20 mg/kg p.o. q24h	Oral and injectable first‐generation fluoroquinolone Although the label dose range is wide, a minimum dose of 10 mg/kg of enrofloxacin and of 5.5 mg/kg of marbofloxacin is recommended for *Staphylococcus* spp. (and 20 mg/kg of enrofloxacin for *Pseudomonas aeruginosa*) High doses should be used if isolates are reported as “SDD” (susceptible dose‐dependent by CLSI) Restricted by regulatory authorities in some countries
Marbofloxacin	2.75–5.5 mg/kg p.o. q24h	
Orbifloxacin	7.5 mg/kg p.o. q24h	Oral third‐generation fluoroquinolone May not be available or be restricted for dogs in all countries
Pradofloxacin	3–6 mg/kg p.o. q24h	Oral third‐generation fluoroquinolone High doses should be used if isolates are reported as “SDD” (susceptible dose‐dependent by CLSI) Restricted by regulatory authorities in some countries
Levofloxacin	25 mg/kg p.o. q24h	Oral third‐generation fluoroquinolone Human‐only fluoroquinolone Should only be considered when veterinary licensed fluoroquinolones are not an option
Doxycycline	5 mg/kg p.o. q12h or 10 mg/kg p.o. q24h	Oral tetracycline May not be available for dogs in all countries Susceptibility testing is advised for the specific drug, not just the class of tetracyclines
Minocycline	5 mg/kg p.o. q12h	Human‐only oral tetracycline Gastrointestinal adverse effects (vomiting) more frequent than with doxycyline May be more active than doxycycline against some strains of *Staphylococcus* spp., but susceptibility testing is advised before considering minocycline.
Trimethoprim‐sulfadiazine or ‐sulfamethoxazole	15 mg/kg p.o. q12h (or 30 mg/kg q24h)	May be considered for dogs in otherwise good general health and if owners have been made aware of a greater risk for clinical adverse effects. These drugs can show good clinical efficacy with a lesser impact on further drug‐resistance No approved susceptibility testing standards and breakpoints available for isolates from animals; human breakpoints and testing standards may be applied using trimethoprim‐sulfamethoxazole Dose‐dependent adverse reactions: keratoconjunctivitis sicca, folate deficiency, drug‐induced hypothyroidism Idiosyncratic reactions/sulfonamide hypersensitivity: overall risk greater than with other antimicrobials, true incidence unclear: fever, blood dyscrasias, polyarthropathy, skin eruptions, acute hepatopathy, (risk may be very low for ormetoprim‐sulfadimethoxine), proteinuria A breed‐associated increased risk may exist for Doberman pinschers but evidence for this remains unclear
Ormetoprim‐sulfadimethoxine	55 mg/kg p.o. loading dose on the first day, then 27.5 mg/kg p.o. q24h	

*Note*: Colours are critically important to highlight the different drug groups according to the antimicrobial group traffic light system.

Abbreviations: CLSI, Clinical Laboratory Standards Institute; p.o., per os (administered orally); q24h, once daily every 24 hours; q12h, twice daily every 12 h.

### Reserved antimicrobial drugs

Drugs listed as ‘Reserved antimicrobial drugs’ should be limited to treating infections caused by multidrug‐resistant staphylococci, mainly MRSP, when no first‐ or second‐choice options are appropriate (Table 5). They are either not authorised or in some countries they are banned from use in dogs because they are critically important for the treatment of serious infections in human medicine. Extended susceptibility testing may have to be requested specifically for each isolate as drugs may not be routinely reported by laboratories. Consideration of the following five criteria before use is strongly recommended:

- Bacterial culture and antimicrobial susceptibility tests from a recent isolate indicate susceptibility, and no other drugs are appropriate.
- Risks for adverse treatment effects have been evaluated after clinical assessment.
- The prognosis for resolving the pyoderma is good.
- Underlying causes have been identified; a treatment plan is in place to prevent recurrences.
- Owners are aware of the risks and need for compliance associated with treatment.

**TABLE 5:** Reserved antimicrobial drugs for canine pyoderma.

Reserved antimicrobial drugs: Only to be considered for infections caused by multidrug‐resistant staphylococci (MRSP, MRSA, MRSC) and after in vitro susceptibility has been confirmed. All with greater concern for significant adverse clinical effects		
**Drug**	**Suggested dosage for dogs**	**Comments**
Rifampicin (Rifampin)	5 mg/kg p.o. q12h or 10 mg/kg p.o. q24h	No veterinary‐label formulations No approved susceptibility testing breakpoints available for isolates from animals; human breakpoints and testing standards may be applied Hepatotoxicity, monitoring of serum biochemistry before treatment and every 7–10 days is recommended May be considered for dogs in otherwise good general health and if owners have been made aware of a greater risk for clinical adverse effects
Amikacin	15 mg/kg i.v., i.m. or s.c. q24h	Possibly good efficacy with lesser impact on further drug‐resistance Approved susceptibility testing standards and breakpoints available for staphylococci from dogs Nephrotoxicity (IRIS http://www.iris‐kidney.com/education/prevention.html May be considered for dogs that are in otherwise good general health and if owners have been made aware of a greater risk for clinical adverse effects. These drugs can show good clinical efficacy with a lesser impact on further drug‐resistance Monitoring of serum biochemistry before treatment and every 7–10 days and of urine every 3–5 days (declining specific gravity (USG) and casts)
Gentamicin	9–14 mg/kg i.v., i.m. or s.c. q24h	
Chloramphenicol	40–50 mg/kg PO q8h	Most *Staphylococcus* spp., including MRSP, will not be susceptible using the new CLSI breakpoints. Many adverse effects are possible in animals, and pet owners should be aware of the risks from exposure to chloramphenicol

*Note*: Colours are critically important to highlight the different drug groups according to the antimicrobial group traffic light system.

Abbreviations: CLSI: Clinical Laboratory Standards Institute; MRSA: meticillin‐resistant *S. aureus*; MRSC: meticillin‐resistant *S. coagulans (*formerly *S. schleiferi*); MRSP: meticillin‐resistant *Staphylococcus pseudintermedius*; P.o.: per os (administered orally); q12h: twice daily every 12 hours; q24h: once daily every 24 h; q8h: three times daily every 8 hours; s.c./i.m./i.v.: administered by subcutaneous/intramuscular/intravenous injection.

### Strongly discouraged drugs

A fourth group of 'Strongly discouraged drugs’ includes linezolid and vancomycin. These drugs are only licensed for use in humans and should be considered ‘reserved’ for the treatment of serious multidrug‐resistant infections in humans. Their use in all animals has been banned in the European Union since 2023.^4^

## PREVENTING RECURRENCES OF PYODERMA

In dogs that require systemic antimicrobial therapy more than once a year for recurrent pyoderma, the search for underlying primary causes must be intensified or repeated.

A thorough diagnostic review of potential primary underlying diseases should be repeated at least every 6–12 months, with the choice of tests guided by the skin lesions and clinical signs that remain after pyoderma has been resolved through successful treatment.

In dogs with recurrent superficial pyoderma secondary to underlying allergic skin disease, appropriate medication or management strategies for their allergy should be prioritised over repeated treatment with antimicrobials.

Allergic, including atopic, skin diseases are suspected and diagnosed clinically if erythema and pruritus remain at predilection sites after ectoparasites and microbial infections have been resolved. The most appropriate drugs and management options for controlling allergic skin disease depend on the clinical signs and stage of disease in each case.^5^

Proactive topical antimicrobial therapy with antiseptics may be effective in preventing relapses and can be maintained indefinitely.

Evidence for efficacy of proactive topical antimicrobial therapy and of the few alternatives such as autogenous *S. pseudintermedius* bacterins, S. *aureus* lysate, bacteriophage therapy, skin probiotics and bacterial interference approaches in preventing recurrences of pyoderma is sparse and new studies are urgently needed.

## METICILLIN‐RESISTANT STAPHYLOCOCCAL PYODERMA

Management concepts and treatment recommendations for surface, superficial, and deep pyoderma apply also to MRS (meticillin‐resistant *S. pseudintermedius, S. aureus* and *S. coagulans*) pyoderma; more detailed advice can be found in MRS‐specific guidelines.^6^

If meticillin‐resistance is reported in coagulase‐negative staphylococci from skin, their clinical relevance may be doubtful and careful consideration of clinical signs and cytology is recommended before antimicrobial therapy is prescribed.

Coagulase‐negative staphylococci are widely distributed as commensals in animals and humans, and multidrug‐resistance, including meticillin‐resistance, is common. However, their clinical relevance needs to be confirmed through review of the clinical findings, cytology and review of sampling and culture method before deciding on the need for antimicrobial treatment.

Topical antimicrobial therapy as the sole antibacterial treatment modality is the treatment of choice for all cases of surface and superficial MRS pyoderma.

If systemic therapy is needed for MRS pyoderma and if susceptibility is reported for non‐beta lactam, first‐choice or second‐choice antimicrobials, these should be prescribed. Adjunctive topical antimicrobial therapy is recommended for every case.

The prognosis for MRS pyoderma is considered good but infection control measures and the potential for zoonotic transmission require attention.

## CONCLUSIONS

Routine use of cytology, topical antimicrobial therapy and a focus on correcting underlying primary causes that lead to pyoderma will substantially reduce our reliance on systemic antimicrobials when managing dogs with pyoderma.

For cases that need systemic antimicrobial therapy, information in the guidelines will help to use antimicrobials responsibly according to current evidence and consensus.

## AUTHOR CONTRIBUTIONS

**Anette Loeffler:** Conceptualization; methodology; data curation; investigation; writing – original draft; validation; writing – review and editing; formal analysis; project administration. **Christine L. Cain:** Conceptualization; methodology; investigation; validation; formal analysis; writing – review and editing. **Lluís Ferrer:** Conceptualization; methodology; investigation; validation; formal analysis; writing – review and editing. **Koji Nishifuji:** Conceptualization; methodology; investigation; validation; formal analysis; writing – review and editing. **Katarina Varjonen:** Conceptualization; methodology; investigation; validation; formal analysis; writing – review and editing. **Mark G. Papich:** Conceptualization; methodology; investigation; validation; formal analysis; writing – review and editing. **Luca Guardabassi:** Conceptualization; methodology; investigation; validation; formal analysis; writing – review and editing. **Siân M. Frosini:** Conceptualization; methodology; investigation; validation; formal analysis; writing – review and editing. **Emi N. Barker:** Conceptualization; methodology; investigation; validation; formal analysis; writing – review and editing. **Scott J. Weese:** Conceptualization; methodology; validation; writing – review and editing.

## FUNDING INFORMATION

Self‐funded.

## CONFLICT OF INTEREST STATEMENT

None of the authors have received any financial contribution for the guideline project.

## Data Availability

The data that support the findings of this study are available from the corresponding author upon reasonable request.
